# Green Phytic Acid-Assisted Synthesis of LiMn_1−x_Fe_x_PO_4_/C Cathodes for High-Performance Lithium-Ion Batteries

**DOI:** 10.3390/nano14161360

**Published:** 2024-08-19

**Authors:** Yueying Li, Chenlu Hu, Zhidong Hou, Chunguang Wei, Jian-Gan Wang

**Affiliations:** 1 School of Energy and Electrical Engineering, Qinghai University, Xi’ning 810016, China; liyy2019@qhu.edu.cn; 2 State Key Laboratory of Solidification Processing, Shaanxi Joint Laboratory of Graphene (NPU), Center for Nano Energy Materials, School of Materials Science and Engineering, Northwestern Polytechnical University, Xi’an 710072, China; 3 School of Renewable Energy, Inner Mongolia University of Technology, Ordos 017010, China

**Keywords:** lithium manganese phosphate, ion doping, phytic acid, lithium-ion batteries

## Abstract

As a promising cathode material, olivine-structured LiMnPO_4_ holds enormous potential for lithium-ion batteries. Herein, we demonstrate a green biomass-derived phytic-acid-assisted method to synthesize a series of LiMn_1−x_Fe_x_PO_4_/C composites. The effect of Fe doping on the crystal structure and morphology of LiMnPO_4_ particles is investigated. It is revealed that the optimal Fe doping amount of x = 0.2 enables a substantial enhancement of interfacial charge transfer ability and Li^+^ ion diffusion kinetics. Consequently, a large reversible capacity output of 146 mAh g^−1^ at 0.05 C and a high rate capacity of 77 mAh g^−1^ at 2 C were acquired by the as-optimized LiMn_0.8_Fe_0.2_PO_4_/C cathode. Moreover, the LiMn_0.8_Fe_0.2_PO_4_/C delivered a specific capacity of 68 mAh g^−1^ at 2 C after 500 cycles, with a capacity retention of 88.4%. This work will unveil a green synthesis route for advancing phosphate cathode materials toward practical implementation.

## 1. Introduction

The utilization of Li-ion batteries (LIBs) as a reliable power source in electric vehicles has aroused great scientific and industrial research interest [1,2,3,4,5]. The employed cathode materials have a vital role in determining their energy/power density and safety. The olivine-type LiFePO_4_ (LFP) has garnered extensive attention in recent decades due to its high theoretical capacity of 170 mAh g^−1^, economically, as well as excellent thermal stability [6,7,8,9,10,11]. However, the relatively low discharge plateau (~3.4 V vs. Li^+^/Li) and the associated low energy density (586 Wh kg^−1^) of LFP impede future applications demanding high energy density. In sharp contrast, the isostructural LiMnPO_4_ (LMP) demonstrates similar capacity output yet with a higher voltage platform (4.1 V vs. Li^+^/Li), leading to a great enhancement of the theoretical energy density by about 20%. Notably, the LMP confronts serious drawbacks of poor electrical conductivity (~10^−10^ S cm^−1^) and small Li-ionic diffusion coefficient (~10^−16^ cm^2^ s^−1^). Moreover, the Jahn–Teller effect of Mn^3+^ during the phase transformation between LMP and MnPO_4_ further results in structural instability [12,13,14,15]. Therefore, the critical problems of LMP should be carefully mitigated before its practical deployment.

A variety of design strategies have been put forward to accelerate the electron transport efficiency and promote the diffuse ability of Li^+^, such as by reducing the particle size [15,16,17], decorating LMP with carbonaceous materials [18,19,20,21,22], and doping/substituting with cations [23,24,25,26,27]. Extraordinarily, substituting the Mn–site with cations (such as Fe^2+^, Mg^2+^, Ni^2+^, Co^2+^) has been reported to effectively stabilize the crystal structure and promote charge transfer through alleviating the Jahn–Teller distortion of Mn^3+^ [28,29]. For example, Liang et al. designed a unique composite of Ti-doped LMP@NaTi_2_(PO_4_)_3_@C/graphene with core double-shell architecture [30]. This composite structure achieves swift bulk ion diffusion and yields a limited capacity fade of about 6.7% after a 600 cycling period. Li et al. fabricated a dual metal-doped Li_1−x_Na_x_Mn_0.8_Fe_0.2_PO_4_/C nanocapsule which also presents improved Li^+^ diffusion capability and excellent durability [31].

Biomass materials that possess the merits of abundance, sustainability, and environmental friendliness have been widely applied to prepare electrode materials. The morphology and properties of cathode materials will be affected by the internal carbon source of biomass materials [32]. Benefiting from the six carbon atoms and six phosphate groups, phytic acid (PhyA) is deemed as an attractive form of phosphorus. Moreover, PhyA is capable of chelating with positively charged multivalent cations [33,34], which is of great benefit for material synthesis. For example, PhyA was employed for the rational synthesis of LiFePO_4_ with superior electrochemical performance [9,34,35]. This inspires us to explore its applicability in LiMnPO_4_-based cathode materials.

In this work, biomass-derived PhyA is utilized as a green phosphorus source to synthesize LiMn_1−x_Fe_x_PO_4_. A series of LiMn_1−x_Fe_x_PO_4_/C are fabricated via the PhyA-assisted solvothermal method following a calcination process. The influence of the Fe^2+^ doping ratio on the crystal structure and electrochemical performance of LMP/C is systematically studied. The LiMn_0.8_Fe_0.2_PO_4_/C exhibits accelerated charge transfer efficiency and enhanced Li-ion diffusion ability, leading to an enhanced cycling and rate capability. A discharge capacity of 133 mAh g^−1^ at 0.05 C after 150 cycles is obtained with a good capacity retention of 91.1%. Additionally, the LMFP/C-0.2 reveals a low capacity loss of 11.4% even after 500 cycles at 2 C, making it a promising cathode candidate for LIBs.

## 2. Materials and Methods

### 2.1. Materials’ Synthesis

The LiMn_1−x_Fe_x_PO_4_/C was synthesized by using LiOH·H_2_O, PhyA, Mn(Ac)_2_, and FeSO_4_·7H_2_O as raw materials. The proportion of Li:(Mn+Fe):P in the precursor was set as 3:1:1.5 in a molar ratio. First of all, LiOH·H_2_O (18 mmol), (Mn(Ac)_2_ + FeSO_4_) (6 mmol), and PhyA (1.5 mmol) were separately dissolved in ethylene glycol (EG, 15 mL) solution under continuous magnetic stirring, forming different starting solutions. The doping ratio of Fe was controlled by altering the molar quantity of FeSO_4_·7H_2_O in the starting solution. Then, the above (Mn(Ac)_2_ + FeSO_4_) and LiOH·H_2_O solutions were added to the PhyA solution successively. Afterwards, the mixture was transferred and sealed in a Teflon-lined stainless-steel autoclave, which was heated to 180 °C for 10 h. The products were collected after being centrifuged, washed with distilled water and ethanol, and dried at 60 °C in a vacuum oven overnight. Finally, the composite was mixed with 20 wt% glucose and heat-treated at different temperatures (350 °C for 2 h and 650 °C for 4 h) under a flow of nitrogen. Eventually, LMFP/C-x (x = 0.1, 0.2, 0.4) was acquired. As a reference, a pure LiMnPO_4_ sample was also synthesized under the same conditions, and named LMP/C.

### 2.2. Material Characterization

X-ray powder diffraction (XRD, X’ Pert Pro MPD, Philips, Almelo, The Netherlands) was employed to detect the crystal structure. The morphology was observed by field-emission scanning electron microscopy (SEM, NanoSEM 450, FEI, Portland, OR, USA) operated at 15 kV. The Brunauer–Emmett–Teller (BET) surface area was acquired by N_2_ adsorption test. The microstructure was examined through a transmission electron microscope (TEM, Tecnai F30G2, FEI, Portland, OR, USA). X-ray photoelectron spectroscopy (XPS, ESCALAB 250Xi, Thermo Scientific, Waltham, MA, USA) was conducted to evaluate the chemical states of different elements. Raman microscopy (Renishaw inVia, Wotton-under-Edge, UK) was employed to study the states of carbon. The carbon content was studied by thermogravimetric analysis (TGA, TGA/DSC3+, Metler Toledo, Switzerland) in the flowing air atmosphere (35 to 880 °C, 10 °C min^−1^).

### 2.3. Electrochemical Measurements

A coin-type (CR2025) half cell was assembled to evaluate the electrochemical behavior of different samples. To obtain the coating paste, the active materials (70 wt%), carbon black (20 wt%), and poly-vinylidene fluoride (PVDF, 10 wt%) binder were mixed in N-methyl-2-pyrrolidone (NMP) solvent. The resulting pastes were pressed on the current collector (aluminum foil) using a doctor blade technique. A Li plate was used for the counter electrodes, along with Celgrade 2400 as the separator and 1 M LiPF6 in ethylene carbonate (EC)/diemethyl carbonate (DMC)/diethyl carbonate (DEC) (1:1:1 by volume) as the electrolyte. Galvanostatic charge/discharge tests were performed on a Land Battery Testing system with a voltage range of 2.5–4.6 V at room temperature. The specific charge and discharge capacities are based on the weight of the LMFP material. A workstation was applied to evaluate the electrochemical performance, including cyclic voltammetry (CV) and electrochemical impedance spectroscopy (EIS). CV plots were acquired within a voltage range of 2.5–4.7 V and a scan range of 0.1–0.5 mV s^−1^. The EIS test frequency limits were set between 0.1 Hz and 100 KHz and the ac signal amplitude was 10 mV.

## 3. Results

**Figure 1:**
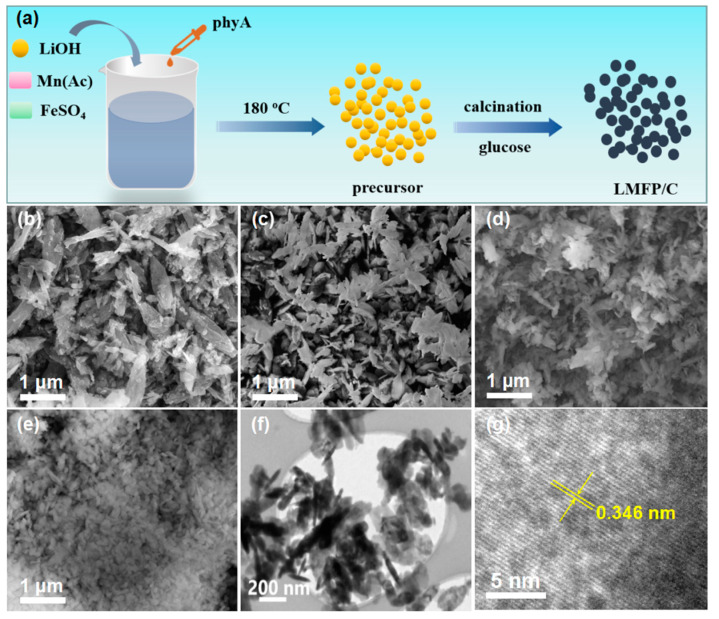
(**a**) Schematic illustration for the fabrication of LMFP/C-x. SEM images of (**b**) LMP/C, (**c**) LMFP/C-0.1, (**d**) LMFP/C-0.2, and (**e**) LMFP/C-0.4. (**f**) TEM and (**g**) HRTEM images of LMFP/C-0.2.

Figure 1a illustrates the fabrication procedure of the LMFP/C-x composite. The LMFP precursor was obtained through a solvothermal process, with PhyA as the green phosphorus source. Subsequently, with the introduction and carbonization of glucose, a homogeneous carbon layer was coated on the surface of LMFP, forming LMFP/C composite. Figure 1b–e display the SEM images of LMFP/C with different Fe doping amounts. Notably, the nanorod morphology of LMP/C ran away gradually and evolved into nanoparticles with increasing Fe doping amount, suggesting that the introduction of Fe can inhibit the crystal growth. The TEM images of the LMFP/C-0.2 (Figure 1f) demonstrate that the LMFP/C-0.2 consists of particles with a size of hundreds of nanometers. A distinct lattice spacing of 0.346 nm corresponds to the characteristic lattice plane of (201) in the HRTEM image of LMFP/C-0.2 [36].

**Figure 2:**
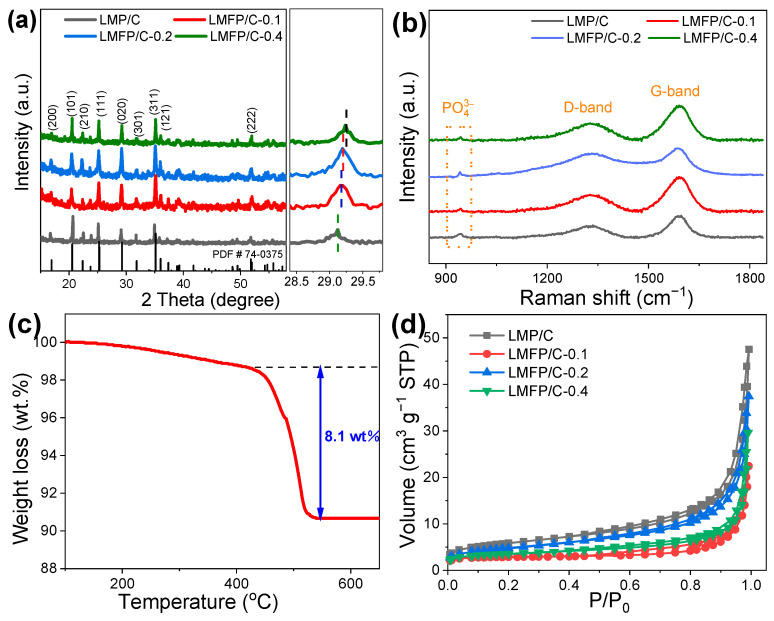
(**a**) XRD patterns and (**b**) Raman spectra of LMP/C, LMFP/C-0.1, LMFP/C-0.2, and LMFP/C-0.4 with an enlarged XRD profile of (020) plane (right part). (**c**) TGA curve of LMFP/C-0.2. (**d**) N_2_ adsorption–desorption isotherms of various samples.

XRD patterns of LMFP/C-x samples are displayed in Figure 2a. Apparently, the major peaks are well indexed as an LMP phase (JCPDS card no. 74-0375) with an olivine-type structure. It is worth pointing out that there is a gradually positive shift of the (020) peak with the increasing of the Fe doping amount, which is related to the minor contraction of the lattice resulting from the differences in the radii of Fe^2+^ (0.65 Å) and Mn^2+^ (0.80 Å). This phenomenon also testifies to the successful doping of Fe element into the LMP lattice. The structural information is further detected by Raman spectra (Figure 2b). The weak characteristic peaks of PO_4_^3−^ centered at ~944 cm^−1^ are monitored in all samples. The other two distinct broad peaks belonging to the disordered carbon (D-, 1340 cm^−1^) and the graphitic carbon (G-, 1590 cm^−1^) testify to the presence of carbon species [13,24,37]. TGA analysis reveals that the carbon content in LMFP/C-0.2 is approximately 8.1 wt% (Figure 2c). The electronic conductivity is believed to be enhanced by the carbon species. The porous structure of LMFP/C-x was characterized by N_2_ adsorption–desorption tests (Figure 2d). According to the IUPAC classification, all samples deliver type IV isotherms with H3 hysteresis loops in the range of 0.5 < P/P_0_ < 1, proving the formation of mesoporous structures [8,35]. The BET specific surface areas of LMP/C, LMFP/C-0.1, LMFP/C-0.2, and LMFP/C-0.4 particles are determined to be 25.6, 15.8, 21.7, and 18.3 m^2^ g^−1^, respectively. The unique porous structure with large specific surface area is beneficial for electron migration and electrochemical reactions.

**Figure 3:**
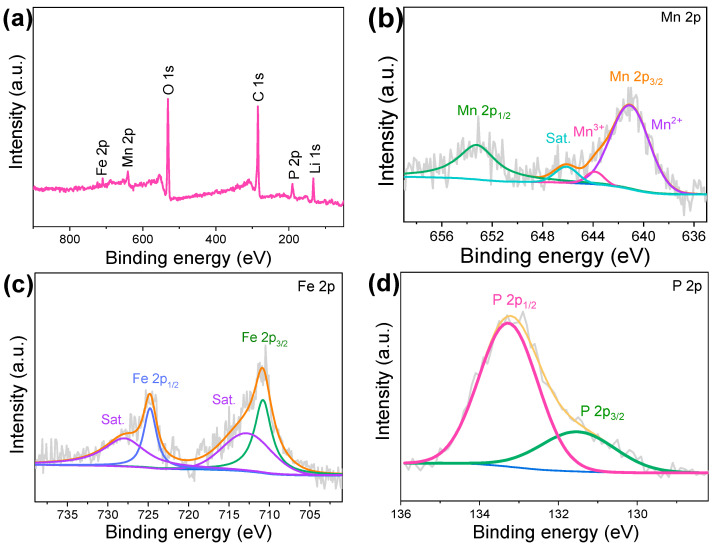
(**a**) XPS survey spectrum of LMFP/C-0.2. High-resolution XPS spectra of (**b**) Mn 2p, (**c**) Fe 2p, and (**d**) P 2p of LMFP/C-0.2.

The chemical states of LMFP/C-0.2 were characterized through XPS measurements. The co-existence of Fe, Mn, Li, P, O, and C elements in the LMFP/C-0.2 confirms the effective doping of Fe element in LMP samples (Figure 3a). Figure 3b displays the fine spectrum of Mn 2p. The fitting peaks with binding energies at 641.0, 643.5, and 653.2 eV are typical of the spin-orbit coupling components of Mn^2+^ 2p_3/2_, Mn^3+^ 2p_3/2_, and Mn^2+^ 2p_1/2_, revealing the hybrid Mn oxidation state of +2/+3 [38]. The trace amount of Mn^3+^ is primarily due to the partial oxidation of Mn^2+^ during high-temperature solvothermal synthesis. The Fe 2p component is characterized with double peaks at 710.9 and 724.7 eV, which belong to the Fe 2p_3/2_ and Fe 2p_1/2_ of Fe^2+^ species (Figure 3c). Figure 3d displays the core-level P 2p, which evidently demonstrates the existence of PO_4_^3−^ groups [30].

**Figure 4:**
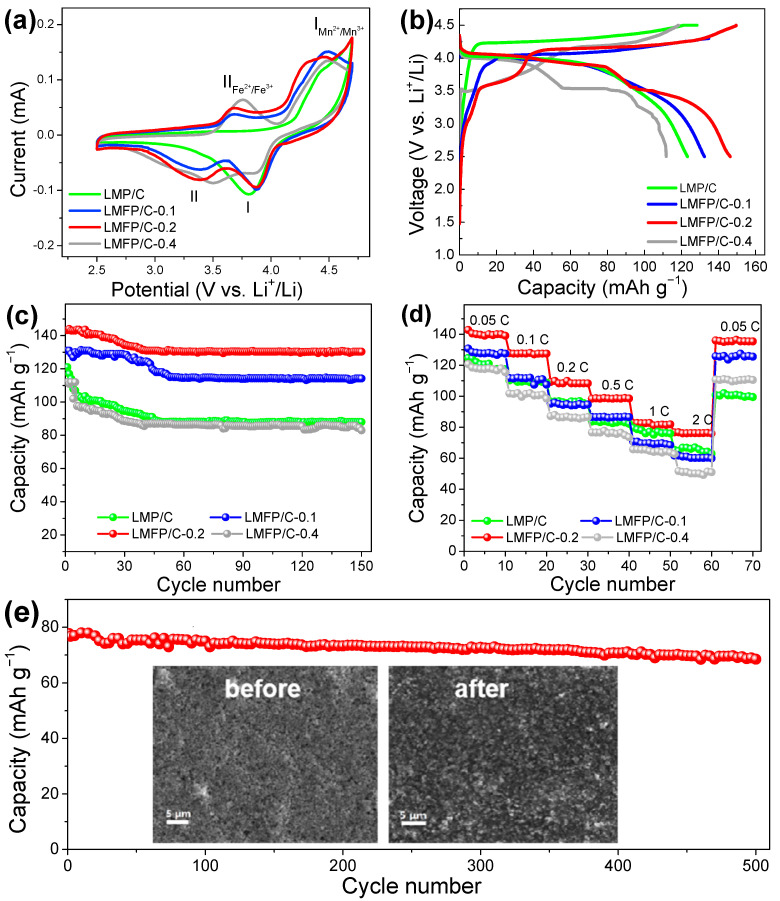
(**a**) CV curves at a scan rate of 0.05 mV s^−1^, (**b**) charge/discharge curves, (**c**) cycling stabilities at 0.05 C, and (**d**) rate performances of LMFP/C, LMFP/C-0.1, LMFP/C-0.2, and LMFP/C-0.4. (**e**) Long-term stability of LMFP/C-0.2 at 2 C (inset: SEM images of the electrode before (**left**) and after (**right**) cycling test).

The electrochemical behavior of the LMFP/C-x cathodes was first monitored by CV measurements (Figure 4a). Compared with pure LMP, two pairs of anodic/cathodic peaks located at about 3.6 V/3.4 V and 4.2 V/3.9 V represent the two-step charge/discharge reactions of the Fe^2+^/Fe^3+^ and Mn^2+^/Mn^3+^ redox couples, respectively. Figure 4b compares the initial charge–discharge profiles of the four samples. It can be seen that a new plateau at about 3.4 V appears in all Fe-doped samples, which brings extra discharge capacity. As a result, the discharge capacity is improved from 123 mAh g-1 (LMP/C) to 132 mAh g^−1^ (LMFP/C-0.1) and 146 mAh g^−1^ (LMFP/C-0.2). This can be attributed to the enhanced intrinsic conductivity and the increased utilization proportion of the active material [35,38]. By contrast, an excessive introduction of Fe^2+^ would induce serious lattice distortion and thus lead to the declined capacity of LMFP/C-0.4 (111.7 mAh g^−1^).

The cycling performance of LMP/C and LMFP/C-x at a 0.05 C rate are displayed in Figure 4c. It can be noted that the discharged capacities of LMFP/C-0.2 only decreased from 146 mAh g^−1^ to 133 mAh g^−1^. The capacity retention (91.1%) was much higher than those of LMP/C (72.7%), LMFP/C-0.1 (86.2%), and LMFP/C-0.4 (74.1%). The enhanced performance was further validated by the rate capabilities. As presented in Figure 4d, the average capacities at 0.05, 0.1, 0.2, 0.5, 1, and 2 C of LLMFP/C-0.2 were 141, 128, 110, 99, 83, and 77 mAh g^−1^, respectively. When tested at 0.05 C again, the specific capacity could recover the initial level, indicating the excellent and durable rate capability of LMFP/C-0.2 under a long-term dynamic cyclic test. More significantly, when cycling at a high-rate of 2 C for 500 cycles (Figure 4e), the LMFP/C-0.2 electrode revealed a low capacity loss of 11.4%. The post-mortem morphological analysis of the cycled electrode clearly substantiated the intact surface without cracks (inset of Figure 4e and Figure S3), which is responsible for the cycling stability caused by Fe doping. It is worth noting that the electrochemical performance of LMFP/C-0.2 not only surpassed that of the pure LMP electrode, but also stood out among the previously reported LMP and LMFP cathodes [39,40].

**Figure 5:**
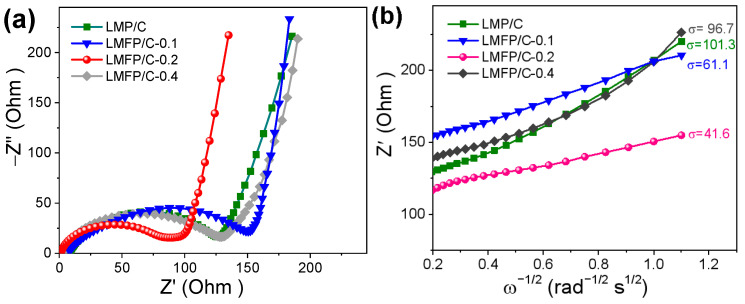
(**a**) Nyquist plots and (**b**) corresponding relationship between Z′ and ω^−1/2^ of the electrodes in the low-frequency region.

To understand in depth the underlying mechanism of the enhanced electrode kinetics, Nyquist plots of LMFP/C-x are measured. As displayed in Figure 5a, all plots consist of a compressed semicircle representing the charge transfer impedance (high-frequency region) and a diagonal line corresponding to the Warburg impedance (low-frequency range). Compared to other electrodes, the LMFP/C-0.2 electrode yielded the smallest diameter of the semicircle (i.e., R_ct_: ~89 Ω) and the steepest slope of the oblique line, illustrating an accelerated charge transfer ability and the Li ions’ diffusion efficiency. The Warburg factor (σ) is related to the Li ion diffusion coefficient (D_Li+_), which can be calculated using the following formula [35]:

D_Li+_ = R^2^T^2^/2n4F^4^A^2^C^2^σ^2^

Here, R, Fc, and T represent the gas constant, the Faraday constant, and the absolute temperature, respectively. n denotes the number of electrons transferred during the reaction. A and C refer to the electrode area and the Li^+^ concentration, respectively. As shown in Figure 5b, the LMFP/C-0.2 shows the smallest σ value of 41.6, much lower than those for the LMP/C (101.3), LMFP/C-0.1 (61.1), and LMFP/C-0.4 (96.7), revealing an improved D_Li+_ (~1.19 × 10^−12^ cm^2^ s^−1^). The combination of lowest Rct and highest D_Li+_ for the LMFP/C-0.2 sample contributes to fast lithiation/delithiation kinetics desirable for the superior rate performance.

## 4. Conclusions and Perspectives

In summary, LMFP/C-x composite materials were successfully synthesized using the green PhyA-assisted solvothermal method. The effect of Fe^2+^ doping was systematically studied, and it was revealed that a certain amount of Fe^2+^ doping enabled a decrease in particle size, an enhancement of charge transfer efficiency, and a promotion of Li^+^ diffusion kinetics. As a consequence, the as-optimized LMFP/C-0.2 sample displayed an elevated initial specific capacity of 146 mAh g^−1^ at 0.05 C, along with a superb rate performance of 77 mAh g^−1^ at 2 C and commendable cycling durability. The present work provides a facile PhyA-assisted synthesis route for developing LiMPO_4_ cathodes, aiming towards high-energy LIBs.

## Data Availability

The data presented in this study are available in the article or Supplementary Materials.

## Supplementary Materials

The following supporting information can be downloaded at: https://www.mdpi.com/article/10.3390/nano14161360/s1, Figure S1: Charge/discharge curves of LMFP/C-0.2 electrode at different cycles (0.05C); Figure S2: Charge/discharge curves of LMFP/C-0.2 electrode at different cycles (2C); Figure S3: Low magnification of LMFP/C-0.2 electrode after cycling test.
