# Supplementary material for: Green Phytic Acid-Assisted Synthesis of LiMn1−xFexPO4/C Cathodes for High-Performance Lithium-Ion Batteries
Source: Nanomaterials (Basel). 2024 Aug 19;14(16):1360. doi: 10.3390/nano14161360 (PMC11357480; doi:10.3390/nano14161360)
Supplement: Supplementary file 1 [file nanomaterials-14-01360-s001.zip › nanomaterials-3122922-supplementary.pdf]

Supporting information for

Green phytic acid-assisted synthesis of $LiMn_{1-x}Fe_xPO_4$/C cathodes for high-performance lithium-ion batteries

Yueying Li [1], Chenlu Hu [2], Zhidong Hou[2], Chunguang Wei[3], and Jian-Gan Wang[1, 2,*]

[1] School of Energy and Electrical Engineering, Qinghai University, Xi'ning 810016, China; liyueying0729@163.com;
[2] State Key Laboratory of Solidification Proces`sing, Center for Nano Energy Materials, School of Materials Science and Engineering, Northwestern Polytechnical University and Shaanxi Joint Lab of Graphene (NPU), Xi'an 710072, China; clhu@mails.nwpu.edu.cn, houzhidong@mails.nwpu.edu.cn;
[3] Shool of Renewable Energy, Inner Mongolia University of Technology, Ordos 017010, China wcg@imut.edu.cn;
* Correspondence: wangjiangan@nwpu.edu.cn;

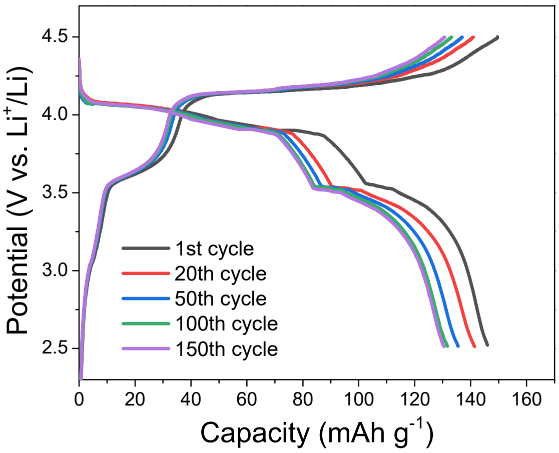

Figure S1 Charge/discharge curves of LMFP/C-0.2 electrode at different cycles (0.05C).

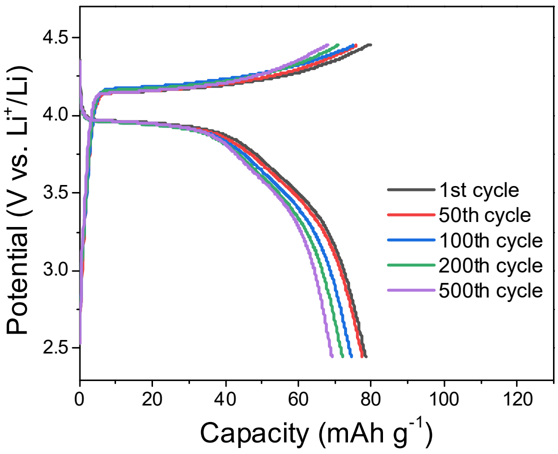

Figure S2 Charge/discharge curves of LMFP/C-0.2 electrode at different cycles (2C).

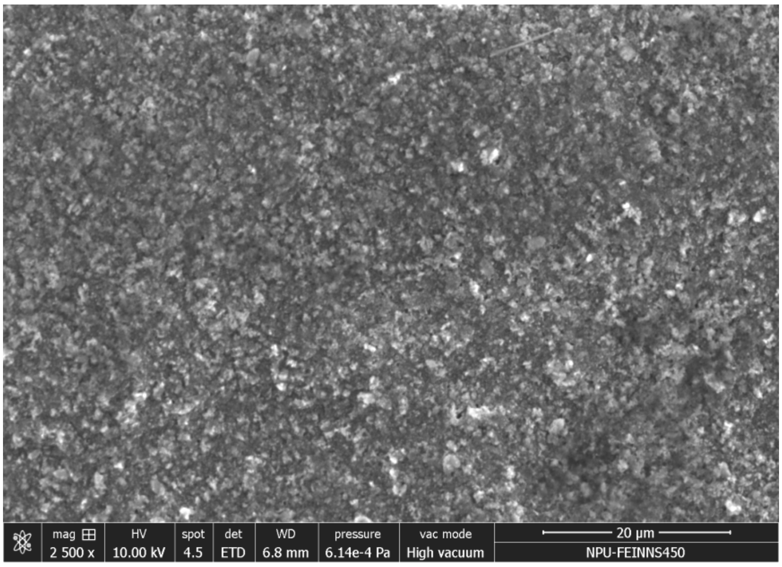

Figure S3 Low magnification of LMFP/C-0.2 electrode after cycling test.
